# Chitosan-Based Hydrogels Containing Nystatin and Propolis as a Novel Tool for *Candida auris* Skin Decolonization

**DOI:** 10.3390/gels11070498

**Published:** 2025-06-26

**Authors:** Andra-Cristina Bostănaru-Iliescu, Andra-Cristina Enache, Ionuț Iulian Lungu, Corneliu Cojocaru, Robert Capotă, Paula Cucu, Maria Liliana Iliescu, Valeria Harabagiu, Mihai Mareș, Alina Stefanache

**Affiliations:** 1 Faculty of Veterinary Medicine, “Ion Ionescu de la Brad” Iasi University of Life Sciences, 8 Mihail Sadoveanu Alley, 700489 Iasi, Romaniapaula.cucu@iuls.ro (P.C.); 2 Academy of Romanian Scientists, Ilfov 3, 050044 Bucharest, Romania; 3 “Petru Poni” Institute of Macromolecular Chemistry, 41A Grigore Ghica Voda Alley, 700487 Iasi, Romania; humelnicu.andra@icmpp.ro (A.-C.E.);; 4 Faculty of Pharmacy, “Grigore T. Popa” University of Medicine and Pharmacy, 16 University Street, 700115 Iasi, Romania; 5 Faculty of General Medicine, “Grigore T. Popa” University of Medicine and Pharmacy, 16 University Street, 700115 Iasi, Romania

**Keywords:** *Candida auris*, nystatin, propolis, chitosan, hydrogels, antifungal activity

## Abstract

*Candida auris* is an emerging multidrug-resistant fungal pathogen with a high affinity for skin colonization and significant potential for nosocomial transmission. This study aimed to develop and evaluate chitosan-based hydrogels loaded with nystatin and propolis as a topical antifungal strategy for skin decolonization of *C. auris*. The formulations were selected based on our previous results and optimized for cutaneous application. The internal structure of the hydrogels was investigated by polarized light microscopy, confirming the amorphous nature of propolis and the partial dispersion of nystatin. The antifungal activity was assessed against ten fluconazole-resistant *C. auris* strains. The CS-NYS-PRO1 formulation demonstrated the highest antifungal performance in the agar test, also reducing viable cell counts to undetectable levels within 6 h. Time–kill assays and SEM imaging confirmed the rapid fungicidal effect and revealed severe membrane disruption and cytoplasmic leakage. Molecular docking analyses indicated the strong binding of nystatin to both sterol 14α-demethylase (CYP51) and dihydrofolate reductase (DHFR) from *C. auris*, suggesting complementary membrane and intracellular mechanisms of action. These findings support the use of such hydrogels as a local, non-invasive, and biocompatible strategy for managing *C. auris* colonization, with promising implications for clinical use in infection control and the prevention of skin-mediated transmission in healthcare settings.

## 1. Introduction

*Candida auris* is an emerging multidrug-resistant fungal pathogen that has become a global concern due to its capacity to persistently colonize the skin and cause life-threatening infections, particularly in immunocompromised individuals [1]. This global concern stems not only from the pathogen’s resistance to multiple antifungal drugs but also from its ability to persist in healthcare settings and its high transmission potential [2]. First identified in 2009 from the ear canal of a patient in Japan, *C. auris* is taxonomically related to *Candida haemulonii* but exhibits distinct clinical and epidemiological features [3]. Since its discovery, *C. auris* has rapidly disseminated across six continents, with outbreaks reported in over 45 countries and high mortality rates from bloodstream infections reaching up to 60% [4].

Skin colonization is considered a major reservoir for this fungal pathogen. Also, *C. auris* is notoriously difficult to eliminate from the skin and mucosal surfaces of colonized individuals, primarily due to its ability to form resilient biofilms. These biofilms persist on abiotic surfaces and are resistant to commonly used antifungals like azoles, echinocandins, and polyenes [5,6,7]. This yeast has been described as a “stealth” or “incognito” pathogen due to its unusual biological traits that make it difficult to detect, treat, and control [8,9]. These include the following: (i) *C. auris* exhibits a preference for skin colonization over the gastrointestinal tract or other anaerobic environments, likely due to its poor growth under anaerobic conditions [10]; (ii) its marked skin tropism enables persistent colonization and rapid patient-to-patient transmission, making it the first fungal pathogen designated as an urgent global public health threat [1,8,10]; (iii) it can survive on abiotic surfaces and medical equipment for prolonged periods, facilitating nosocomial outbreaks even under standard cleaning protocols [11,12,13]; (iv) diagnostic laboratories using conventional phenotypic or biochemical assays frequently misidentify *C. auris*, delaying appropriate intervention [14]; (v) the pathogen exhibits resistance to multiple major classes of antifungal agents, with bloodstream infections associated with mortality rates between 30 and 60% [4,15]. For example, in the U.S., for instance, the number of *C. auris* skin colonization cases nearly tripled between 2020 and 2021, while invasive infections almost doubled [16].

**Figure 1:**
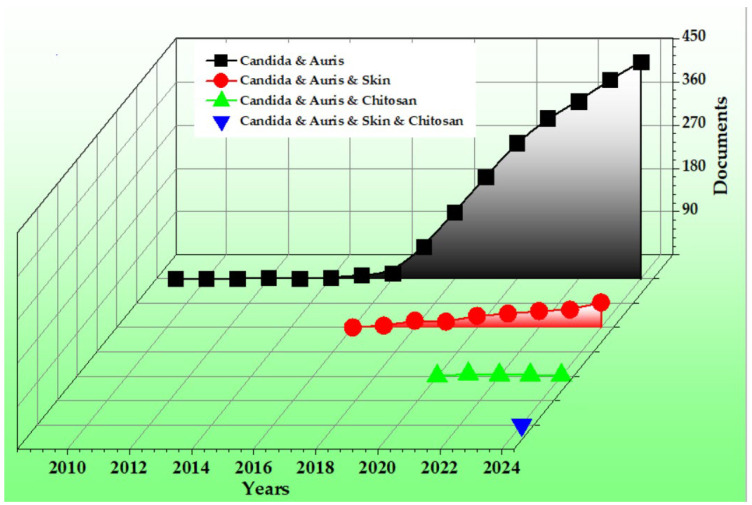
Analysis of research trends on *Candida auris* skin infections and chitosan-based approaches (Scopus-indexed documents containing the keywords “*Candida*”, “*auris*”, “*skin*”, and/or “*chitosan*” in the title, abstract, or keyword fields; search conducted on 28 May 2025).

Although research on *Candida auris* has expanded rapidly since its initial identification, rising from a single publication in 2009 to over 450 in 2024, less than 10% of studies have focused on its skin colonization stage, which is critical for transmission (Figure 1).

Despite numerous alternative antifungal strategies being explored, including antifungal combinations [17,18] drug repurposing, and the development of novel antifungal agents such as small molecules [19,20], polymers [21], and natural product derivatives [22], as well as innovative approaches like nanoparticles, hydrogels, antimicrobial coatings, and irradiation techniques [21,23], no universally effective treatment has been approved. This underscores the urgent need for biocompatible delivery technologies that can overcome fungal resistance and environmental persistence.

Among such systems, chitosan-based hydrogels have attracted considerable interest for combating *Candida* species. Derived from chitin, chitosan is biocompatible, biodegradable, mucoadhesive, and intrinsically antimicrobial, making it an ideal for localized, controlled-release formulations [24]. Its gel-forming ability under mildly acidic conditions allows for the encapsulation and sustained release of hydrophobic antifungals like nystatin [25]. Furthermore, chitosan hydrogels exhibit favorable physicochemical properties for skin delivery systems, including strong bioadhesiveness and controlled drug release [26].

Our previous work demonstrated that chitosan-based films and hydrogels loaded with nystatin (NYS) and/or propolis (PRO) exhibit strong antifungal activity against *Candida albicans* and *Candida glabrata*, while maintaining suitable mechanical properties for mucocutaneous application [27]. Moreover, Enache et al. investigated the release of insoluble nystatin from physically and chemically modified chitosan hydrogels in simulated fluids, reporting a pronounced antifungal activity against *Candida albicans*, *Candida dubliniensis*, and *Candida glabrata* [28]. Nystatin was also shown to enhance hydrogel stability through hydrophobic and hydrogen bonding interactions [28], while propolis a natural resinous compound collected by bees, with antimicrobial and anti-inflammatory properties may exert synergistic effects with NYS, allowing for lower effective doses [27].

However, as shown in Figure 1, few researchers have explored the potential use of chitosan in *Candida auris* skin decolonization [29]. This highlights a significant research gap, particularly in light of the urgent need for effective topical formulations capable of disrupting colonization and reduce the risk of nosocomial spread. The scarcity of studies focused on skin-targeted, chitosan-based delivery systems for *C. auris* underscores the novelty and relevance of the current investigation.

This study is, to our knowledge, the first to evaluate chitosan-based hydrogels containing both nystatin and propolis as a targeted strategy for the topical decolonization of *Candida auris*. Building upon our previous work on chitosan-based films and hydrogels incorporating nystatin and/or propolis previously developed for vulvovaginal and oral candidiasis [27], here we focus exclusively on hydrogel formulations, optimized for cutaneous application and specifically tailored to address a critically underexplored infection model.

Compared to solid films, hydrogels offer superior hydration, enhanced skin adhesion, and a microenvironment favorable to both antifungal activity and tissue healing [30]. However, current antifungal treatments, such as commercial nystatin-based ointments (e.g., Nidoflor^®^, Mycolog-II^®^), often contain synthetic excipients (e.g., corticosteroids, parabens, alcohols) that may cause local irritation or allergic reactions, particularly in sensitive patients. Accordingly, this work proposes a novel, well-tolerated strategy for managing *C. auris* colonization by harnessing chitosan’s functional properties and the antifungal synergy of nystatin and propolis in a biocompatible hydrogel matrix. In addition, to gain deeper mechanistic insights into the antifungal action of this formulation, molecular docking analyses were conducted to investigate nystatin interactions with key *Candida auris* enzymes, including sterol 14-alpha demethylase (CYP51), a well-known target involved in ergosterol biosynthesis. Notably, this study is the first to explore the potential binding of nystatin to dihydrofolate reductase (DHFR), an essential enzyme in folate metabolism and nucleic acid synthesis, suggesting a possible alternative antifungal mechanism.

## 2. Results and Discussion

### 2.1. Hydrogel Preparation and Microstructural Analysis

The preparation and characterization of chitosan-based hydrogels incorporating nystatin and propolis were initially established in our previous work [27], focusing on antifungal activity against *Candida albicans* and *Candida glabrata*. These formulations were extensively characterized in the form of films (morpho-structural and mechanical properties) as well as in gel form (rheological properties, dynamic vapor sorption) for their antifungal activity, relevant to muco-cutaneous candidiasis, with perspectives to demonstrate the additive or synergistic effect of these two active principles against other *Candida* species. Notably, rheological analysis confirmed that gels containing higher amounts of propolis exhibited predominantly viscous behavior, while those with chitosan and/or nystatin alone showed solid-like characteristics [27].

**Figure 2:**
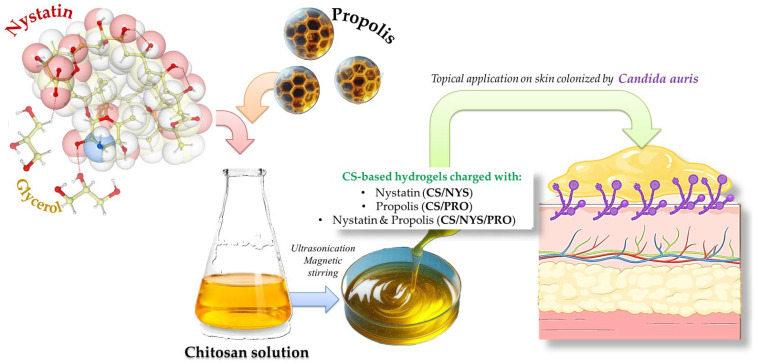
Schematic representation of CS-based hydrogels loaded with nystatin and/or propolis preparation (CS/Nys, CS/Pro, and CS/Nys/Pro) as novel tool for *Candida auris* skin decolonization.

Building on this foundation, the current study adapts the hydrogel preparation method, to further explore chitosan-based hydrogels incorporating nystatin and propolis, or topical applications targeting *Candida auris* skin decolonization. Thus, chitosan-based hydrogels containing nystatin (CS-NYS), propolis (CS/PRO), or both active agents in different ratios (CS/NYS/PRO1, respectively, CS/NYS/PRO2) were prepared, with slight adaptations tailored to topical applications. A schematic representation of the hydrogel preparation process used here is presented in Figure 2. This approach ensures reproducibility while allowing us to investigate additional structural and functional properties relevant for skin application and antifungal efficacy against this emerging pathogen.

**Figure 3:**
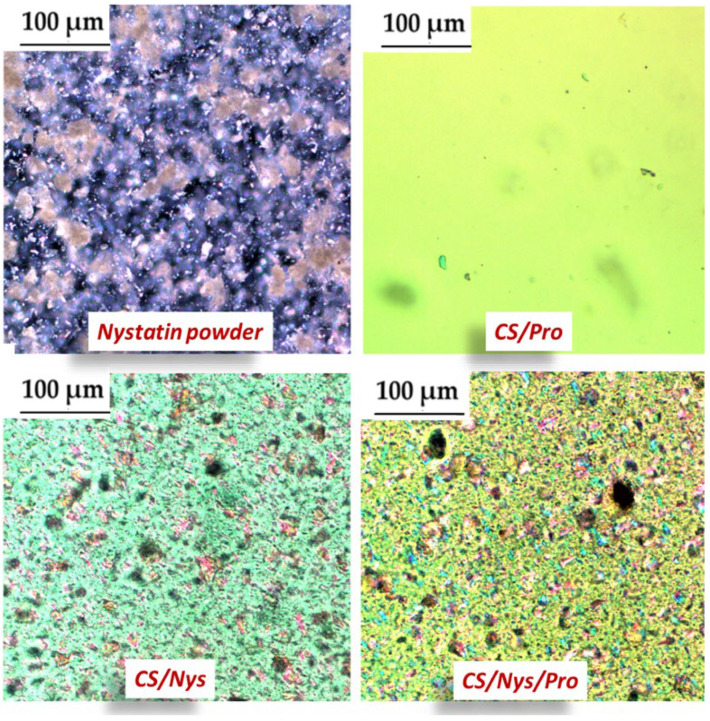
Polarized light microscopy (PLM) images of CS-based hydrogels showing microstructural differences related to composition (nystatin powder, chitosan hydrogel loaded with propolis, and/or nystatin).

The microstructural analysis of CS/Nys, CS/Pro, and CS/Nys/Pro hydrogels was performed using polarized light microscopy (PLM), in comparison with pure nystatin powder, as illustrated in Figure 3. This technique enables the visualization of anisotropic structures and crystalline domains through their birefringence patterns.

Nystatin, a polyene antifungal with low water solubility [31,32], typically exists in crystalline form. Therefore, under PLM, nystatin crystals exhibit characteristic birefringence due to their ordered internal lattice, appearing as bright white structures (Figure 3). This behavior has been previously reported in studies of solid-state nystatin formulations, confirming its crystalline nature [27,33].

Upon incorporation into the chitosan hydrogel matrix (CS/NYS), these particles exhibit birefringent coloration, suggesting interactions with the polymeric network and partial microscale dispersion. This observation aligns with previous findings regarding nystatin’s low solubility and crystalline morphology [27,28].

In contrast, CS/PRO displays a homogeneous, amorphous texture with no visible birefringent particles, consistent with the known amorphous nature of propolis extracts. The CS/NYS/PRO hydrogel presents a hybrid microstructure, in which birefringent crystalline particles coexist with amorphous regions. This reflects the combined influence of nystatin and propolis on the internal microstructural organization of the gel matrix.

Moreover, PLM analysis indicates a generally uniform distribution of nystatin within the hydrogel formulations, supporting its successful incorporation into the polymeric matrix despite its intrinsic crystallinity. These microstructural variations may influence the antifungal activity of the formulations, which is further evaluated through biological assays against *Candida auris*. In addition, molecular docking studies can provide insights into the potential interaction mechanisms between nystatin and fungal targets.

### 2.2. Evaluation of the Antifungal Activity of Hydrogel Formulations Against Candida auris

The in vitro antifungal activity of the chitosan-based hydrogel formulations was evaluated against a panel of ten *Candida auris* strains (CBS 10913, CBS 18248–18256), using the agar disk diffusion method. After 48 h of incubation at 37 °C, antifungal efficacy was determined by measuring the diameter of the clear zones of inhibition surrounding each hydrogel.

**Figure 4:**
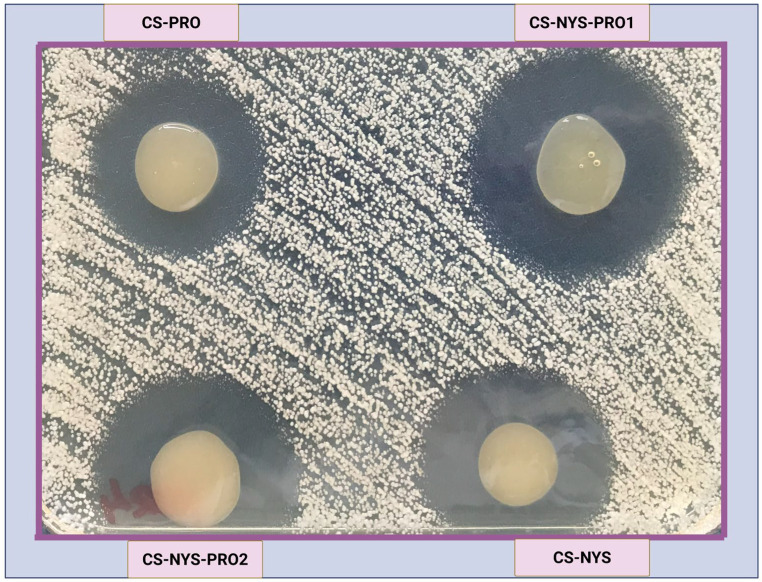
Representative agar disk diffusion image showing the inhibition zones produced by the hydrogel formulations against *C. auris*.

As illustrated in Figure 4, all hydrogels exhibited antifungal activity in direct contact with *C. auris* colonies. However, measurable zones of inhibition extending into the surrounding agar were observed mainly for the nystatin-containing hydrogels (CS-NYS, CS-NYS-PRO1, and CS-NYS-PRO2). Among these, CS-NYS-PRO1 exhibited the largest visible inhibition zone, followed by CS-NYS-PRO2 and CS-NYS, while the CS-PRO formulation (containing only propolis) showed more limited antifungal activity.

The diameters of the inhibition zones across the tested strains (CBS 10913, CBS 18248, CBS 18249, CBS 18250, CBS 18251, CBS 18252, CBS 18253, CBS 18254, CBS 18255, CBS 18256) are presented in Table 1, along with the corresponding means and standard deviations. CS-NYS-PRO1 showed inhibition zones ranging from 35 to 39 mm, with a mean diameter of 37.5 ± 1.18 mm. CS-NYS-PRO2 ranged from 32 to 36 mm (mean = 34.5 ± 1.51 mm), while CS-NYS showed moderate activity (26–30 mm; mean = 28.3 ± 1.34 mm). The CS-PRO formulation exhibited the smallest inhibition zones (22–26 mm; mean = 23.9 ± 1.29 mm). Statistical analysis (one-way ANOVA, OriginPro v8.5) confirmed significant differences among the formulations (F = 210.36, *p* < 0.0001). High dataset consistency was indicated by an R^2^ value of 0.946 and a coefficient of variation of 4.2%.

Specifically, CS-NYS-PRO1 exhibited the largest diameter (39 mm) against strain CBS 18251 and maintained strong activity (≥38 mm) against the majority of strains (CBS 10913, CBS 18248, CBS 18249, CBS 18250, CBS 18255, and CBS 18256). CS-NYS-PRO2 exhibited slightly reduced, but still notable, inhibition values (up to 36 mm across four C. auris strains), while CS-NYS provided only moderate inhibition. The limited efficacy of CS-PRO confirms the critical role of nystatin in the formulation. The presence of nystatin appears to be essential for achieving effective antifungal activity beyond the hydrogel–colony interface, and the enhanced performance of CS-NYS-PRO1 may result from improved diffusion and synergistic effects.

Although some variability in inhibition zone diameters was observed among the *C. auris* strains (Table 1), no clear correlation could be established between strain identity and antifungal susceptibility under the test conditions. This suggests that the differences in inhibition may reflect minor phenotypic variations rather than significant resistance heterogeneity. Therefore, all tested strains exhibited similar sensitivity trends, further supporting the robustness of the hydrogel formulations.

The antifungal activity results of the four hydrogel formulations (CS-NYS, CS-PRO, CS-NYS-PRO1, and CS-NYS-PRO2) against *Candida auris* were aggregated for each hydrogel formulation. Figure S1 provides a visual comparative summary of the inhibition zone diameters for each hydrogel formulation, highlighting the statistically significant differences in antifungal efficacy. The thick horizontal bars represent the mean inhibition diameters.

The graph clearly illustrates the superior performance of CS-NYS-PRO1 (mean diameter: 37.5 mm), followed by CS-NYS-PRO2 (34.5 mm). In contrast, CS-NYS and CS-PRO exhibited lower activity, with mean inhibition zones of 28.3 mm and 23.9 mm, respectively. Thus, Figure S1 complements the numerical data presented in Table 1 and reinforces the conclusion that the nystatin–propolis combination significantly enhances antifungal activity.

These findings highlight the enhanced antifungal performance of the CS-NYS-PRO1 hydrogel, likely due to the synergistic interaction between nystatin and propolis, and support its potential use for the topical decolonization of *Candida auris*.

Compared to other antifungal hydrogels, CS-NYS-PRO formulations demonstrate improved activity. For instance, Pandian et al. (2021) [29] reported inhibition zones of only 20–25 mm against *C. auris*, using octenidine-loaded chitosan dressings. Perchyonok et al. tested various nystatin-containing chitosan hydrogels and observed inhibition zones between 7.8 mm and 10 mm against *Candida albicans*, while pure nystatin alone reached approximately 20 mm [25]. However, the combination of nystatin and propolis showed inhibition zones generally below 25 mm against *C. albicans* and *C. glabrata* [27]. These findings further underscore the superior efficacy of CS-NYS-PRO1 against *C. auris*, a more resistant species, highlighting the therapeutic potential of combining natural bioactive agents with conventional antifungal drugs in advanced topical formulations.

Although no direct comparison with commercial antifungal products was performed in this study, many of which contain synthetic excipients associated with irritation, the chitosan–propolis–nystatin combination offers a promising, naturally derived alternative. Future studies, including time–kill assays and molecular interaction analyses, are warranted to further evaluate its therapeutic potential.

### 2.3. Evaluation of Time–Kill Assay Test

**Figure 5:**
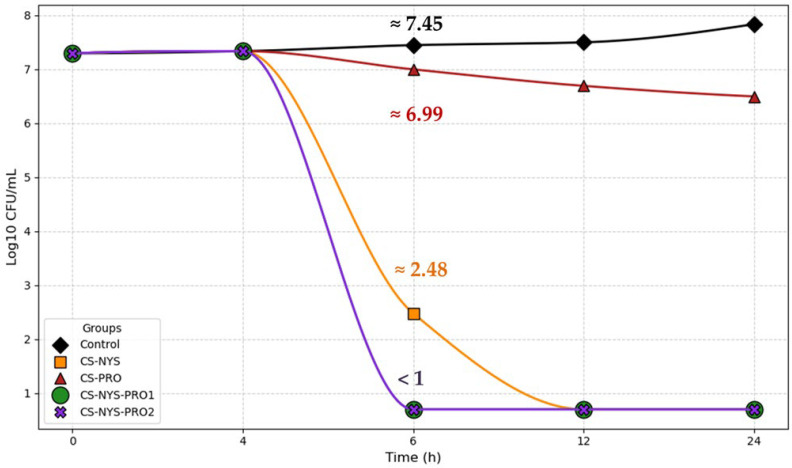
Time–kill curves for evaluation of the fungicidal activity of CS-NYS, CS-PRO, CS-NYS-PRO1, and CS-NYS-PRO2 against yeast cells of *Candida auris* CBS 10913 compared to the control. The graph highlights the rapid reduction in viable cell counts in 6 h, as CS-NYS-PRO1 and CS-NYS-PRO2 decreased fungal levels from the control value (7.45 log_10_, approximately 2.8 × 10^7^ CFU/mL) to below the detection limit (<1 log_10_ CFU/mL, corresponding to fewer than 10 cells per mL).

The time–depletion kinetics against *Candida auris* CBS 10913 were evaluated for all hydrogel formulations tested in the disk diffusion assays (CS-NYS, CS-PRO, CS-NYS-PRO1, and CS-NYS-PRO2), in comparison with a control sample. The time–kill curves are shown in Figure 5 using a logarithmic scale (log_10_ CFU/mL) to accurately reflect the wide range of fungal viability over time. For clarity, the exact numerical values at key time points are provided in Table S1.

As shown in Figure 5 and Table S1, at 6 h, the control sample exhibited a slight increase in fungal load (log_10_(2.8 × 10^7^) ≈ 7.45 CFU/mL), whereas CS-NYS and CS-PRO hydrogels showed moderate reductions (log_10_(3.0 × 10^2^) ≈ 2.48 and log_10_(9.9 × 10^6^) ≈ 6.99 CFU/mL, respectively). Notably, CS-NYS-PRO1 and CS-NYS-PRO2 achieved near-complete fungal eradication, with counts dropping below the detectable limit (log_10_(<10) < 1 CFU/mL). This corresponds to a >6-log_10_ reduction (over one million-fold decrease) in viable fungal cells for the combined formulations compared to the control. CS-NYS alone reduces counts by approximately 5-log_10_, while CS-PRO exhibits a modest ~0.5-log_10_ reduction. These results highlight the superior fungicidal efficacy of the combined CS-NYS-PRO hydrogels relative to their individual components and the untreated control.

At 24 h, both CS-NYS-PRO1 and CS-NYS-PRO2 maintained their potent fungicidal effects, keeping fungal counts below the detection limit (<10 CFU/mL). CS-NYS also sustained strong activity, reducing viable cells to undetectable levels by 12 h and maintaining this through 24 h. In contrast, CS-PRO showed only a modest reduction (~1–2 log_10_ decrease) in fungal load even after 24 h. Meanwhile, the control sample continued to exhibit exponential fungal growth, reaching 6.79 × 10^7^ CFU/mL by the 24 h mark.

Notably, both chitosan hydrogels loaded with nystatin and propolis demonstrated rapid and potent fungicidal activity against *Candida auris*, outperforming previously reported time–kill assay results for this pathogen. Standard echinocandins (anidulafungin, caspofungin, micafungin) have been shown to be essentially non-fungicidal against *Candida auris* in vitro, failing to achieve ≥3-log_10_ (99.9%) CFU reductions in time–kill assay [34,35]. Amphotericin B, a more commonly used polyene than nystatin, has been reported to cause only transient CFU reductions, with regrowth observed at 12 h even at twice the MIC [36], or to show no significant inhibition after 24 h [37]. Moreover, amphotericin B required higher concentrations and prolonged exposure (~9.13 h) to achieve fungicidal effects [34].

In contrast, the combination of nystatin with propolis embedded in a chitosan matrix achieved the complete killing of *Candida auris* within 6 h, demonstrating unusually rapid and sustained fungicidal efficacy, with viable cell counts dropping below detectable levels.

The exceptional kill kinetics are particularly significant given the notorious multidrug resistance of *C. auris*. This yeast can survive under harsh conditions and is often resistant to azoles, amphotericin B, and even echinocandins [10,15]. Thus, achieving a ≥6-log_10_ reduction in viable *C. auris* with a topical CS-NYS-PRO1 or CS-NYS-PRO2 hydrogel underscores the effectiveness of the combined formulation.

While both formulations were highly effective in the time–kill assay, the performance of CS-NYS-PRO1 is particularly noteworthy when considered alongside the results of the inhibition zone assays (Table 1, Figure S1), where it exhibited the largest inhibition diameter despite containing less propolis than CS-NYS-PRO2. This apparent paradox is consistent with our previous findings in *C. albicans* [27], where a higher propolis content was shown to reduce the in vitro release efficiency of nystatin, as crosslinking between polyphenols and chitosan hinders the diffusion of NYS through the resulting polymeric network. Moreover, the structural and functional advantages of a lower propolis content, also supported by rheological behavior, led to a reduction in fungal loads from approximately 1.24 × 10^7^ CFU/mL to undetectable levels (<10 CFU/mL) for *C. albicans* [27].

Based on these findings, CS-NYS-PRO1 appears to offer a more balanced and effective formulation with clear translational relevance, supporting its potential development as a decolonization or infection-prevention agent in clinical settings. Consequently, the morphological changes induced in *C. auris* by this formulation were further investigated using scanning electron microscopy (SEM).

### 2.4. Evaluation of Morphological Changes in C. auris byScanning Electron Microscopy (SEM)

**Figure 6:**
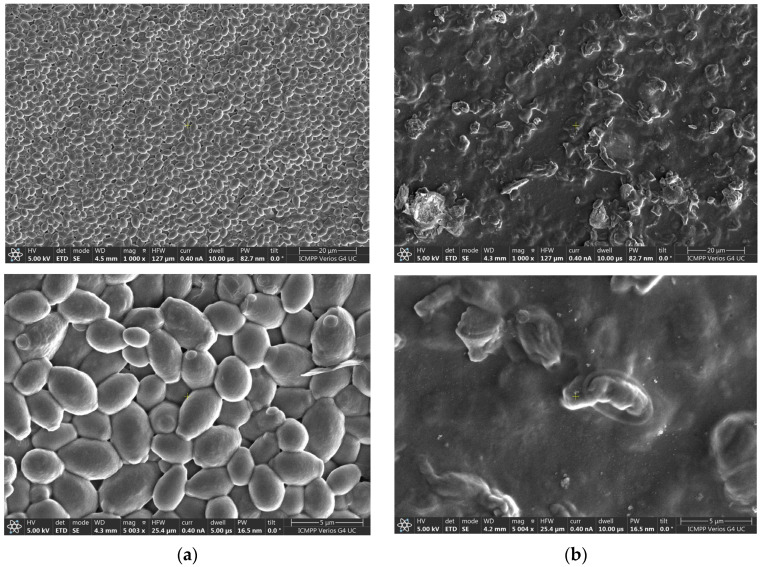
Visualization of the effects of CS-NYS-PRO1 hydrogel on *Candida auris* using advanced scanning electron microscopy (SEM). (**a**) SEM micrographs of untreated *C. auris* cells showing well-defined, smooth surfaces with typical oval morphology; (**b**) *C. auris* cells after treatment with CS-NYS-PRO1 hydrogel displaying aberrant morphology and impaired cell separation.

To further investigate the morphological impact of hydrogel treatment on *Candida auris*, high-resolution scanning electron microscopy (SEM) analysis was performed on CBS 10913 strain cells before and after exposure to the CS-NYS-PRO1 hydrogel. Figure 6a shows untreated *C. auris* cells from the control sample, which display the typical oval yeast morphology with well-defined, smooth surfaces and clear cell boundaries, indicative of healthy, intact membranes, as also reported by others [38]. In contrast, Figure 6b presents *C. auris* cells treated with the CS-NYS-PRO1 hydrogel, revealing pronounced structural alterations. These include irregular and collapsed cell morphologies, surface roughening, and inhibited cell separation. Most notably, the leakage of cytoplasmic content was observed in multiple cells, suggesting severe membrane disruption.

These morphological aberrations indicate that the hydrogel compromises membrane integrity and cellular architecture, likely due to the combined antifungal mechanisms of nystatin targeting ergosterol and propolis, which may enhance oxidative stress and increase membrane permeability.

To strengthen the SEM-based observations, we performed a quantitative analysis using Fiji/ImageJ coupled with Trainable Weka Segmentation, as detailed in Figure S2 in the Supplementary Material. The image analysis approach, including segmentation and quantitative particle analysis, was adapted from the method described by Vyas et al. (2016), which demonstrated biofilm removal efficiency quantification using SEM and image analysis [39].

In untreated *Candida auris* samples, cellular structures occupied 83.91% of the image area (total area = 256,562 pixels^2^; average object size = 64,140.5 pixels^2^), with regular, well-defined morphology. Conversely, samples treated with the CS-NYS-PRO1 hydrogel exhibited a drastically reduced cell-occupied area of only 3.64% (total area = 9784 pixels^2^; average object size = 3.6 pixels^2^), indicative of extensive cellular fragmentation and disruption. Perimeter measurements also decreased significantly, further confirming the loss of cellular integrity. Quantitative analysis thus supports the antifungal efficacy of the CS-NYS-PRO1 hydrogel by revealing over a 95% reduction in cell area (calculated using Equation (S1) in the Supplementary Materials), reflecting significant structural damage and impaired cell morphology following treatment.

In addition, these quantitative SEM outcomes are consistent with the significant reduction in fungal viability observed in both agar diffusion and time–kill assays, reinforcing the hypothesis that the CS-NYS-PRO1 hydrogel induces rapid membrane damage and cellular disintegration in *C. auris*. Thus, SEM findings provide direct ultrastructural evidence supporting the antifungal efficacy of this chitosan-based hydrogel formulation and its potential application in topical decolonization strategies against multidrug-resistant fungal pathogens.

These findings are in line with previously reported effects of polyene antifungals, such as nystatin, which bind in the fungal membrane, forming size-selective pores through hydrophobic interactions between the lipophilic segment of nystatin and the sterol, leading to the loss of cytoplasmic integrity [40]. To further support the mechanistic understanding at the molecular level, docking studies were performed.

### 2.5. Molecular Docking Simulations of Nystatin Binding Mechanisms

As reported by Sousa et al. [40], in addition to their well-established membrane-disruptive activity, polyene antifungals such as nystatin may also act by inducing oxidative stress, DNA damage, and protein and lipid oxidation. These findings suggest that intracellular targets might contribute to their antifungal mechanism. Therefore, to gain deeper mechanistic insights, molecular docking analyses were conducted for nystatin with both sterol 14-alpha demethylase (CYP51), a key enzyme involved in ergosterol biosynthesis [41], and dihydrofolate reductase (DHFR) from *Candida auris*, an essential enzyme in folate metabolism and nucleic acid synthesis [42].

#### 2.5.1. In Silico Analysis of Nystatin Interaction with Ergosterol Biosynthesis Enzyme (CYP51)

As polyene antifungals, azoles, and allylamines exert their antifungal activity by interfering with ergosterol biosynthesis at different stages [40], the crystal structure sterol 14-alpha demethylase (CYP51) from *Candida albicans* (PDB ID: 5FSA) was retrieved from the Protein Data Bank and used to assess the potential binding interactions of nystatin with this membrane-associated enzyme. As detailed in Table 2, the structural characterization of sterol 14-alpha demethylase catalytic domain (CYP51, PDB ID: 5FSA) using YASARA Structure software revealed a protein composed of 484 amino acid residues, with a molecular weight of 55.6 kDa and a radius of gyration (Rg) of 22.82 Å. The solvent-accessible surface area was calculated to be 20,512 Å^2^. The amino acid composition showed a predominance of leucine (8.5%), lysine (7.2%), and threonine/serine (6.6% each), with a nearly balanced net charge (−1), resulting from a close ratio of positively (Lys, Arg) and negatively (Glu, Asp) charged residues. Secondary structure analysis indicated a dominant α-helical content (47.5%), followed by random coils (30.8%), β-sheets (12.6%), turns (8.1%), and a minor presence of 3_10_-helices (1.0%).

Molecular docking simulations were carried out using AutoDock Vina within the YASARA Structure software. A total of 100 docking runs were performed for nystatin (ligand) against the catalytic domain of sterol 14-alpha demethylase receptor (PDB ID: 5FSA). The results yielded 19 distinct binding poses, clustered based on a 5.0 Å RMSD threshold.

**Figure 7:**
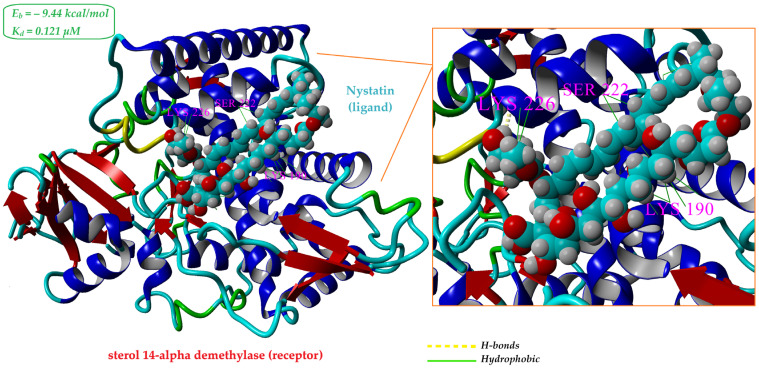
Best docking pose of the receptor (PBD ID: 5FSA) and ligand (nystatin). The ligand (nystatin) is shown with atom-specific coloring: carbon (cyan), nitrogen (blue), oxygen (red), and hydrogen (gray). The receptor’s secondary structure is displayed with α-helices and 3_10_-helix in blue, β-sheets in red, and turns in green and random coils in cyan.

The best docking conformation, representing the most favorable interaction mode, is illustrated in Figure 7. It involved extensive interactions with 25 amino acid residues, including ASN187, MET189, LYS190, GLU194, PHE213, ARG215, SER216, ALA218, GLN219, SER222, ASP225, LYS226, GLY227, PHE228, THR229, PRO230, ILE231, HIS310, ASP504, SER507, MET508, LEU511, PRO512, THR513, and GLU514. Nystatin exhibited a calculated binding energy (Eb) of −9.436 kcal/mol and an estimated dissociation constant (Kd) of 0.121 µM, indicating a strong and stable interaction with the receptor, supporting the high affinity of nystatin for the receptor.

According to the docking analysis (Figure 7), nystatin interacts with 25 amino acid residues on the receptor primarily through hydrophobic contacts. This supports the hypothesis reported in the literature that nystatin forms size-selective pores via hydrophobic interactions between its lipophilic segments and sterol molecules, ultimately causing loss of cytoplasmic integrity [40].

Additionally, Table S2 in the Supplementary Materials, which summarizes the interaction descriptors obtained from the in silico molecular docking between nystatin (ligand) and the CYP51 receptor (PDB ID: 5FSA), shows that alongside 22 hydrophobic contacts, nystatin also forms 3 hydrogen bonds (inset in Figure 7), reflecting a strong and stable binding. Hydrogen bond analysis reveals three key hydrogen bonds that stabilize the complex: between nystatin’s nitrogen and the oxygen atom of LYS226 (2.22 Å, 3.27 kcal/mol), and between nystatin’s oxygen and the oxygen atoms of ASP504 (2.31 Å, 1.87 kcal/mol) and SER507 (2.41 Å, 1.94 kcal/mol). The total hydrogen bond energy sums to 7.09 kcal/mol, indicating a significant contribution of these bonds to the overall ligand–receptor interaction. Notably, all three hydrogen bonds are accepted by receptor atoms, with none donated by the ligand.

These extensive hydrophobic and hydrogen bonding interactions observed in the best docking conformation strongly support the high binding affinity and specificity of nystatin for the CYP51 active site. Given that CYP51 plays a critical role in ergosterol biosynthesis, essential for fungal cell membrane integrity [41], these findings provide a molecular basis for the antifungal efficacy of nystatin observed in our experimental assays. Moreover, the stable binding mode suggests that nystatin effectively inhibits CYP51 function, disrupting ergosterol production and contributing to membrane destabilization and fungal cell death.

#### 2.5.2. In Silico Insights into the Interaction of Nystatin with *Candida auris* Dihydrofolate Reductase

Although nystatin is traditionally recognized for its membrane-targeting antifungal mechanism, we explored the possibility of alternative or complementary mechanisms involving intracellular targets. Dihydrofolate reductase (DHFR) from *Candida auris* (PDB ID: 7ZZX), an enzyme critical for folate metabolism and nucleic acid synthesis, represents a validated antifungal target [42]. Therefore, DHFR was selected as a model receptor to investigate potential binding interactions with nystatin.

The structural and compositional features of crystal structure of *Candida auris* DHFR (PDB ID: 7ZZX) were determined through in silico analysis using the YASARA Structure software (version v.20.8.23) and are summarized in Table S3, in the Supplementary Materials. The balanced distribution of positively and negatively charged residues (net charge = 0) may contribute to the electrostatic neutrality and structural stability of the receptor. Furthermore, the coexistence of polar and non-polar amino acid residues supports the potential for diverse interactions, including hydrogen bonds and hydrophobic contacts. The high proportion of ordered secondary structures (36.6% β-sheets and 23.3% α-helices) underlines the receptor’s conformational rigidity, advantageous for reliable molecular docking simulations.

**Figure 8:**
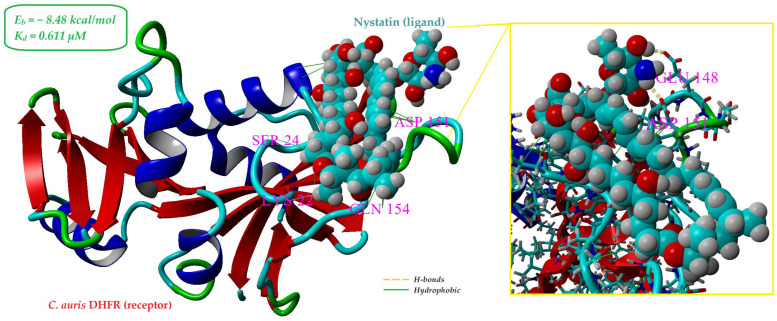
Best docking pose of the receptor (PDB ID: 7ZZX) and ligand (nystatin). The ligand (nystatin) is shown with atom-specific coloring: carbon (cyan), nitrogen (blue), oxygen (red), and hydrogen (gray). The receptor’s secondary structure is displayed with α-helices in blue, β-sheets in red, and turns in green and random coils in cyan.

Molecular docking simulations generated 100 binding poses using AutoDock Vina. The best-scoring pose, based on binding energy and spatial orientation within the DHFR active site, is shown in Figure 8. Nystatin exhibited a calculated binding energy (*E_b_*) of −8.48 kcal/mol and an estimated dissociation constant (*K_d_*) of 0.611 µM, indicating a relatively strong and stable interaction with the receptor.

In this complex, nystatin forms multiple stabilizing interactions with twenty-one amino acid residues, including two hydrogen bonds: one between its polar head and the side chain of ASP151 (2.15 Å) and another with GLU148 (2.24 Å), as evidenced in the inset of Figure 8. Additionally, 16 hydrophobic contacts with an average length of 4.23 ± 0.40 Å further stabilize the complex. These results support the hypothesis that nystatin may interact directly with DHFR and potentially inhibit its enzymatic function.

Molecular docking results (binding energy and dissociation constant) suggest that nystatin binds more strongly to sterol 14-alpha demethylase (CYP51) compared to dihydrofolate reductase (DHFR). Although the affinity for DHFR is slightly lower, it still indicates a relatively strong and stable interaction, suggesting that nystatin may exert its antifungal effects through multiple targets. Compared to classical antifolate inhibitors such as pyrimethamine and cycloguanil—known to exert potent DHFR inhibition [34], nystatin demonstrates a comparable binding affinity. While classical antifolates inhibit DHFR through direct competition at the active site, nystatin is traditionally known for disrupting fungal membranes. Thus, alongside the strong binding affinity observed for CYP51, the notable interaction of nystatin with DHFR suggests that its antifungal activity may involve multiple mechanisms, both at the membrane level and intracellularly. This dual-target potential could enhance the overall efficacy of the CS-NYS-PRO1 hydrogel against multidrug-resistant *Candida auris* strains, offering a broader mechanistic basis for its antifungal properties.

This hypothesis introduces an intriguing perspective on nystatin’s antifungal action and warrants further investigation. While the docking results are promising, in vitro and in vivo experiments are essential to validate the biological relevance and therapeutic implications of this potential DHFR-targeting mechanism.

## 3. Conclusions

Chitosan-based hydrogels with nystatin and/or propolis were structurally characterized, confirming the partial dispersion of nystatin and the amorphous nature of propolis within the formulations. The CS-NYS-PRO hydrogels exhibited a microstructure combining both crystalline and amorphous regions, which may contribute to a synergistic effect at the microstructural level.

The bio-evaluation of the hydrogels confirmed their significant antifungal activity against a diverse panel of fluconazole-resistant *C. auris* strains. Among all tested variants, CS-NYS-PRO1 demonstrated the most pronounced fungicidal effect, as reflected by the largest inhibition zones in disk diffusion assays and the rapid reduction in viable fungal cells in time–kill studies. Within 6 h of exposure, viable counts dropped to below detectable levels (<10^1^ CFU/mL), indicating a rapid and sustained killing effect. These data strongly suggest a synergistic antifungal mechanism between nystatin and propolis when co-delivered in a chitosan matrix.

Scanning electron microscopy of treated *C. auris* cells revealed extensive morphological damage, including membrane disruption, cytoplasmic leakage, and abnormal cell shapes, which corroborate the rapid fungicidal kinetics observed. Such ultrastructural alterations provide strong evidence for the combined physical and chemical modes of action of the hydrogel components. Together, the in vitro mycological results validate the antifungal performance of the CS-NYS-PRO hydrogels and highlight their potential utility in skin decolonization applications. Importantly, the formulations exhibited strong bioactivity under skin-mimicking conditions, making them promising candidates for use in topical therapies to manage *C. auris* colonization and prevent transmission in high-risk clinical environments.

Molecular docking studies revealed that nystatin binds strongly to *Candida auris* sterol 14-alpha demethylase (CYP51), a key enzyme in ergosterol biosynthesis, and also interacts notably with dihydrofolate reductase (DHFR), an essential enzyme involved in folate metabolism and DNA synthesis. Although the binding affinity for DHFR is slightly lower than for CYP51, it remains relatively strong, suggesting a possible complementary intracellular mechanism of antifungal action alongside the well-established membrane-disruptive effects of nystatin. This dual-target potential represents a novel insight into the multifaceted antifungal activity of nystatin, potentially enhancing its efficacy against multidrug-resistant *C. auris* strains.

Future work should aim to evaluate the hydrogels’ performance in more complex in vivo or ex vivo skin models and assess their safety, retention, and efficacy profiles under real-use conditions. Additionally, formulation optimization, including drug-loading capacity and rheological behavior, may further improve clinical applicability. Overall, these findings support the continued investigation of chitosan-based hydrogel platforms as a non-invasive strategy to combat resistant fungal pathogens on skin surfaces.

## 4. Materials and Methods

### 4.1. Materials

Chitosan (Merck Chemical, Saint Louis, MO, USA), L-(+)-lactic acid and glycerin (Chemical Company SA, Iasi, Romania), nystatin (Antibiotice SA, Iasi, Romania), and aqueous propolis extract (Dapis Transilvania, Cluj-Napoca, Romania) were used as received without further purification.

### 4.2. Microbial Strains

The in vitro antifungal activity of chitosan-based hydrogels containing nystatin and/or propolis was evaluated against a panel of ten *Candida auris* strains provided by the Fungal Biodiversity Centre (CBS, Utrecht, The Netherlands): CBS 10913, CBS 18248, CBS 18249, CBS 18250, CBS 18251, CBS 18252, CBS 18253, CBS 18254, CBS 18255, and CBS 18256. Strain identification and characterization were confirmed using DNA sequencing and MALDI-TOF MS analysis. These strains represent diverse genotypic backgrounds and are known to be fluconazole resistant, making them suitable for assessing alternative antifungal therapies.

The hydrogels were tested for efficacy using standard in vitro antifungal activity assays. All experiments were performed in triplicate under controlled laboratory conditions.

### 4.3. Methods

#### 4.3.1. Chitosan-Based Hydrogels Preparation and Characterization

Chitosan-based hydrogels were prepared by adapting previously reported protocols. [27,28]. Briefly, a 3% (*w*/*v*) chitosan stock solution was prepared by dissolving medium molecular weight chitosan in an aqueous lactic acid solution under constant stirring. Nystatin powder (dispersed in glycerin) and propolis were separately incorporated into the chitosan solution under mild conditions to preserve the bioactivity of both compounds. For the combined formulations (CS-NYS-PRO1 and CS-NYS-PRO2), the active ingredients were added in two different mass ratios (PRO/NYS = 3:1 and 6:1, respectively). Each formulation was homogenized and allowed to gel under controlled conditions. Polarized light microscopy (Leica Microsystems Polarized Optical Microscope DM2500 M, Wetzlar, Germany) was employed to investigate the microstructural differences among the hydrogels prepared with various active components.

#### 4.3.2. Antifungal Activity of Hydrogel Formulations Against *Candida auris*

In this study, the antifungal activity of four chitosan-based hydrogel formulations (CS-NYS, CS-PRO, CS-NYS-PRO1, and CS-NYS-PRO2) was investigated against a panel of ten *Candida auris* strains (CBS 10913, CBS 18248–CBS 18256). These strains, all fluconazole-resistant, were selected due to their clinical relevance in both invasive candidiasis and persistent skin colonization. Each isolate was stored in 20% glycerol at −80 °C and reactivated by subculturing on Sabouraud Dextrose Agar (SDA, Biokar Diagnostics, Allonne, France), followed by incubation at 37 °C for 48 h. Standardized yeast suspensions were prepared in sterile saline solution, with turbidity adjusted to match the 0.5 McFarland standard (1 × 10^8^ CFU/mL). A volume of 0.2 mL from each suspension was spread uniformly onto Yeast Nitrogen Base agar (YNB, Merck Chemical, Saint Louis, MO, USA) supplemented with dextrose, pre-poured into sterile Petri dishes. After surface drying, 10 μL of each hydrogel formulation was applied directly to the agar. Plates were incubated at 37 °C for 48 h. Antifungal activity was assessed by measuring the diameter of the inhibition zones formed around the hydrogel application sites. All tests were performed in triplicate under identical conditions to ensure reproducibility. Zone diameters were measured using a calibrated digital caliper.

This method involved the direct application of chitosan-based hydrogels onto dextrose-supplemented YNB agar plates previously inoculated with standardized microbial suspensions (~1 × 10^8^ CFU/mL).

#### 4.3.3. Time–Kill Assay Using Skin-Mimicking Conditions

The time–kill kinetics of the tested chitosan-based hydrogel formulations were investigated against *Candida auris* CBS 10913 using a modified version of synthetic vaginal simulant medium (SVSM), adapted in-house to mimic the physicochemical characteristics of skin colonization sites and simulate cutaneous physiological conditions. This medium provided a biomimetic environment suitable for assessing antifungal performance on skin-colonizing pathogens.

Prior to testing, *C. auris* CBS 10913 was subcultured twice on Sabouraud Dextrose Agar (SDA) and incubated at 37 °C for 24 h to ensure a fresh, active population. A 5 McFarland suspension (1.24 × 10^7^ CFU/mL), standardized using a TC20 automated cell counter (Bio-Rad, Hercules, CA, USA), was prepared in medium. Equal volumes of this yeast suspension and each hydrogel formulation were mixed to create the test mixtures. A negative control consisting of yeast suspension mixed with medium (without hydrogel) was also included. The test and control tubes were incubated at 36 ± 1 °C. At predetermined time intervals (0, 4, 6, 12, and 24 h), 1 mL aliquots were collected, serially diluted in sterile distilled water, and plated on YPD agar. Plates were incubated for 48 h at 36 ± 1 °C, and colony-forming units (CFU/mL) were counted. The detection limit for viable cells was 10^1^ CFU/mL.

All experiments were conducted in duplicate and independently repeated to ensure reproducibility. Time–kill curves (log_10_ CFU/mL versus time) were generated to visualize and compare the fungicidal activity of each hydrogel formulation.

#### 4.3.4. Scanning Electron Microscopy (SEM) of *Candida auris*

For high-resolution ultrastructural imaging, *Candida auris* CBS 10913 was cultured in Sabouraud Dextrose Broth at 37 °C for 24 h under gentle agitation (75 rpm). Following incubation, cells were harvested and washed three times in sterile phosphate-buffered saline (PBS). To assess the ultrastructural effects of treatment, the cells were incubated with the CS-NYS-PRO1 hydrogel for 24 h at 37 °C under gentle agitation (75 rpm). Untreated cells served as the negative control. After treatment, cells were washed with PBS and fixed in 3% glutaraldehyde for 1 h at room temperature. Fixed cells were subsequently washed again in PBS and dehydrated through a graded ethanol series (25%, 50%, 70%, 95%, and 100%), with each step lasting 10 min, except for the final immersion in absolute ethanol, which was extended to 20 min.

After dehydration, samples were coated with a 10 nm platinum layer using a Leica EM ACE200 Sputter Coater (Leica Microsystems, Vienna, Austria). SEM imaging was conducted using a Verios G4 UC Scanning Electron Microscope (Thermo Scientific, Waltham, MA, USA) operated in high vacuum mode. Morphological analysis was performed using a secondary electron detector (Everhart–Thornley Detector, ETD, Thermo Fisher Scientific, Waltham, MA, USA) at an accelerating voltage of 5 kV. Elemental analysis, where applicable, was performed using an integrated Octane Elect Super SDD detector (AMETEK, Tokyo, Japan).

This methodology enabled the detailed visualization of the cell surface morphology of *C. auris* CBS 10913, facilitating structural comparisons between untreated and hydrogel-treated cells.

SEM images were analyzed using Fiji/ImageJ software (v1.54p, National Institutes of Health, Bethesda, MD, USA). The Trainable Weka Segmentation plugin (University of Waikato, Hamilton, New Zealand) was applied to segment the cell structures from the background based on user-trained classifiers. The resulting binary masks were thresholded and analyzed using the Analyze Particles tool from Fiji software to quantify morphological parameters such as total area, average size, and perimeter of cells and cellular fragments. These quantitative metrics were used to assess the extent of cellular damage following treatment with the CS-NYS-PRO1 hydrogel.

#### 4.3.5. Molecular Docking Simulation

Molecular docking simulations were performed on a Dell Precision T7910 workstation with 32 CPU threads, using the AutoDock Vina algorithm as implemented in the YASARA Structure software package (version 20.8.23, YASARA Biosciences GmbH, Vienna, Austria) [43,44]. Two target receptors from *Candida auris* were selected for *in silico* analysis: sterol 14α-demethylase (CYP51; PDB ID: 5FSA) and dihydrofolate reductase (DHFR; PDB ID: 7ZZX). Their three-dimensional structures were retrieved from the Protein Data Bank (PDB). The ligand nystatin was obtained from the PubChem database (CID: 6433272). For CYP51 receptor, the structure was first cleaned using the Yasara Structure program by removing crystallized solvent molecules and native co-crystallized ligands and adding missing hydrogen atoms, thereby retaining only the protein structure. Also, the geometry of nystatin was subjected to energy minimization using YASARA’s molecular mechanics force field to ensure a stable conformation before docking.

The docking simulations yielded 100 possible binding poses; the highest-ranked pose was then selected based on its favorable binding energy and proper orientation within the receptor’s active site. Ultimately, these in silico results complement the experimental findings by providing molecular-level evidence supporting the antifungal efficacy of the nystatin-loaded chitosan formulations against *Candida auris*.

## Figures and Tables

**Table 1:** Inhibition zone diameters (mm) for each chitosan-based hydrogel formulation against ten *Candida auris* strains, with determined means ± standard deviations.

	Inhibition Zone Diameter (mm)			
Strain No.	CS-NYS	CS-PRO	CS-NYS-PRO1	CS-NYS-PRO2
CBS 10913	29	25	38	36
CBS 18248	27	22	38	32
CBS 18249	28	24	38	36
CBS 18250	26	24	38	36
CBS 18251	27	25	39	34
CBS 18252	29	23	37	34
CBS 18253	29	22	36	33
CBS 18254	30	26	35	33
CBS 18255	28	24	38	36
CBS 18256	30	24	38	35
* Mean ± SD	28.3 ± 1.34	23.9 ± 1.29	37.5 ± 1.18	34.5 ± 1.51

* Statistical analysis performed using one-way ANOVA (OriginPro v8.5).

**Table 2:** Main characteristics of sterol 14-alpha demethylase (CYP51) catalytic domain receptor (PDB ID: 5FSA), determined through structural analysis using YASARA Structure software.

Characteristics	Value
Number of amino acid residues	484
Molecular weight (kDa)	55.608
Radius of gyration *R_g_* (Å)	22.82
Solvent accessible surface *SAS* (Å^2^)	20,512
Primary structure content/Amino acid composition (codes):	Count (relative frequency)
Lysine (Lys, K)	35 (7.2%)
Arginine (Arg, R)	23 (4.7%)
Glutamic acid (Glu, E)	30 (6.2%)
Aspartic acid (Asp, D)	29 (6.0%)
Threonine (Thr, T)	32 (6.6%)
Histidine (His, H)	13 (2.7%)
Alanine (Ala, A)	23 (4.7%)
Serine (Ser, S)	32 (6.6%)
Methionine (Met, M)	12 (2.5%)
Proline (Pro, P)	29 (6.0%)
Glycine (Gly, G)	30 (6.2%)
Valine (Val, V)	30 (6.2%)
Cysteine (Cys, C)	4 (0.8%)
Tyrosine (Tyr, Y)	25 (5.2%)
Asparagine (Asn, N)	17 (3.5%)
Isoleucine (Ile, I)	27 (5.6%)
Leucine (Leu, L)	41 (8.5%)
Tryptophan (Trp, W)	7 (1.4%)
Glutamine (Gln, Q)	14 (2.9%)
Phenylalanine (Phe, F)	31 (6.4%)
Sum of positively charged residues, Σ (Lys^+^ + Arg^+^)	+58
Sum of negatively charged residues, Σ (Glu^−^ + Asp^−^)	−59
Net charge of the receptor	−1
Secondary structure content:	(relative frequency)
α-helix	47.5%
β-sheet	12.6%
turn	8.1%
random coil	30.8%
3_10_-helix	1.0%
π-helix	0.0%

## Data Availability

The original contributions presented in this study are included in the article/Supplementary Material. Further inquiries can be directed to the corresponding author(s).

## Supplementary Materials

The following supporting information can be downloaded at: https://www.mdpi.com/article/10.3390/gels11070498/s1. Figure S1: Antifungal activity of the four hydrogel formulations (CS-NYS, CS-PRO, CS-NYS-PRO1, and CS-NYS-PRO2) against *Candida auris*; Table S1: Time-kill efficacy of CS-NYS, CS-PRO, CS-NYS-PRO1 and CS-NYS-PRO2 hydrogels, against *Candida auris* CBS 10913 over 24 h, compared to the control; Figure S2: Workflow for SEM image processing and quantitative analysis using Trainable Weka Segmentation and ImageJ; Table S2: Interaction descriptors from in silico molecular docking between the ligand (nystatin molecule) and the receptor (PDB ID: 5FSA); Table S3: Main characteristics of the receptor (crystal structure of dihydrofolate reductase (DHFR) from the emerging pathogenic fungus *Candida auris* (PDB ID: 7ZZX)), determined through structural analysis using YASARA Structure software.
