# Supplementary material for: Chitosan-Based Hydrogels Containing Nystatin and Propolis as a Novel Tool for Candida auris Skin Decolonization
Source: Gels. 2025 Jun 26;11(7):498. doi: 10.3390/gels11070498 (PMC12294422; doi:10.3390/gels11070498)
Supplement: Supplementary file 1 [file gels-11-00498-s001.zip › gels-3705186-supplementary.pdf]

Supplementary material

Article

Chitosan-Based Hydrogels Containing Nystatin and Propolis as a Novel Tool for *Candida auris* Skin Decolonization

Andra-Cristina Bostănaru-Iliescu, Andra-Cristina Enache, Ionut Iulian Lungu, Corneliu Cojocaru, Robert Capotă, Paula Cucu, Maria Liliana Iliescu, Valeria Harabagiu, Mihai Mareș, Alina Stefanache

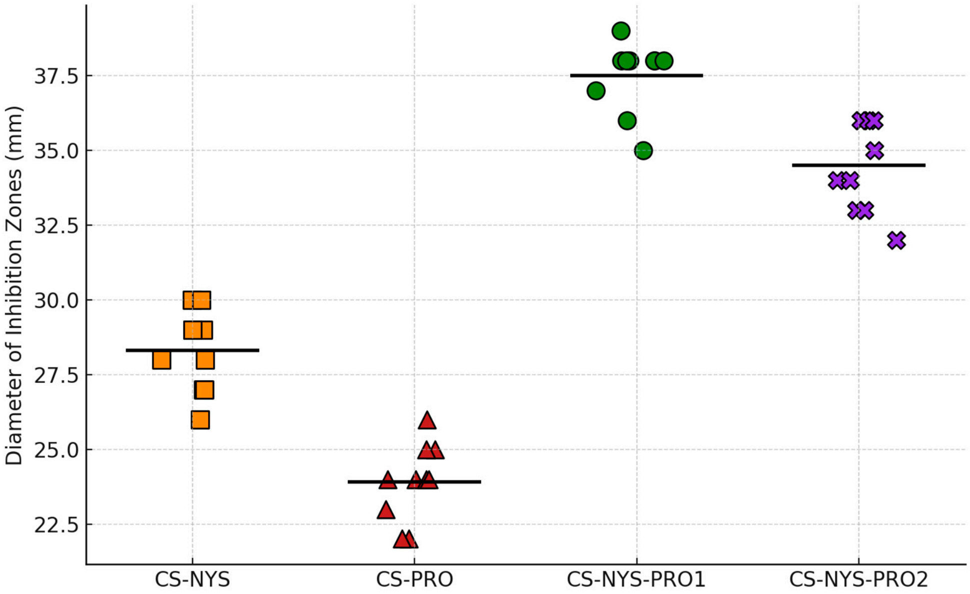

Figure S1. Antifungal activity of the four hydrogel formulations (CS-NYS, CS-PRO, CS-NYS-PRO1, and CS-NYS-PRO2) against Candida auris. Each symbol represents the inhibition zone diameter measured for one of the ten tested strains. Thick horizontal bars represent the mean values. Statistically significant differences were confirmed using one-way ANOVA ($F = 210.36$, $p < 0.0001$).

Table S1. Killing-time efficiency of CS-NYS, CS-PRO, CS-NYS-PRO1 and CS-NYS-PRO2 hydrogels, against *Candida auris* CBS 10913 in 24 h as compared to the control test.

Time (hours)	*Candida auris* (CFU/mL)				
	Control	CS-NYS	CS-PRO	CS-NYS-PRO1	CS-NYS-PRO2
0	$1.96x10^{7}$	$1.96x10^{7}$	$1.96x10^{7}$	$1.96x10^{7}$	$1.96x10^{7}$
4	$2.15x10^{7}$	$2.15x10^{7}$	$2.15x10^{7}$	$2.15x10^{7}$	$2.15x10^{7}$
6	$2.78x10^{7}$	$3.0x10^{2}$	$9.89x10^{6}$	$<10^{1}$	$<10^{1}$
12	$3.13x10^{7}$	$<10^{1}$	$4.89x10^{6}$	$<10^{1}$	$<10^{1}$
24	$6.79x10^{7}$	$<10^{1}$	$3.12x10^{6}$	$<10^{1}$	$<10^{1}$

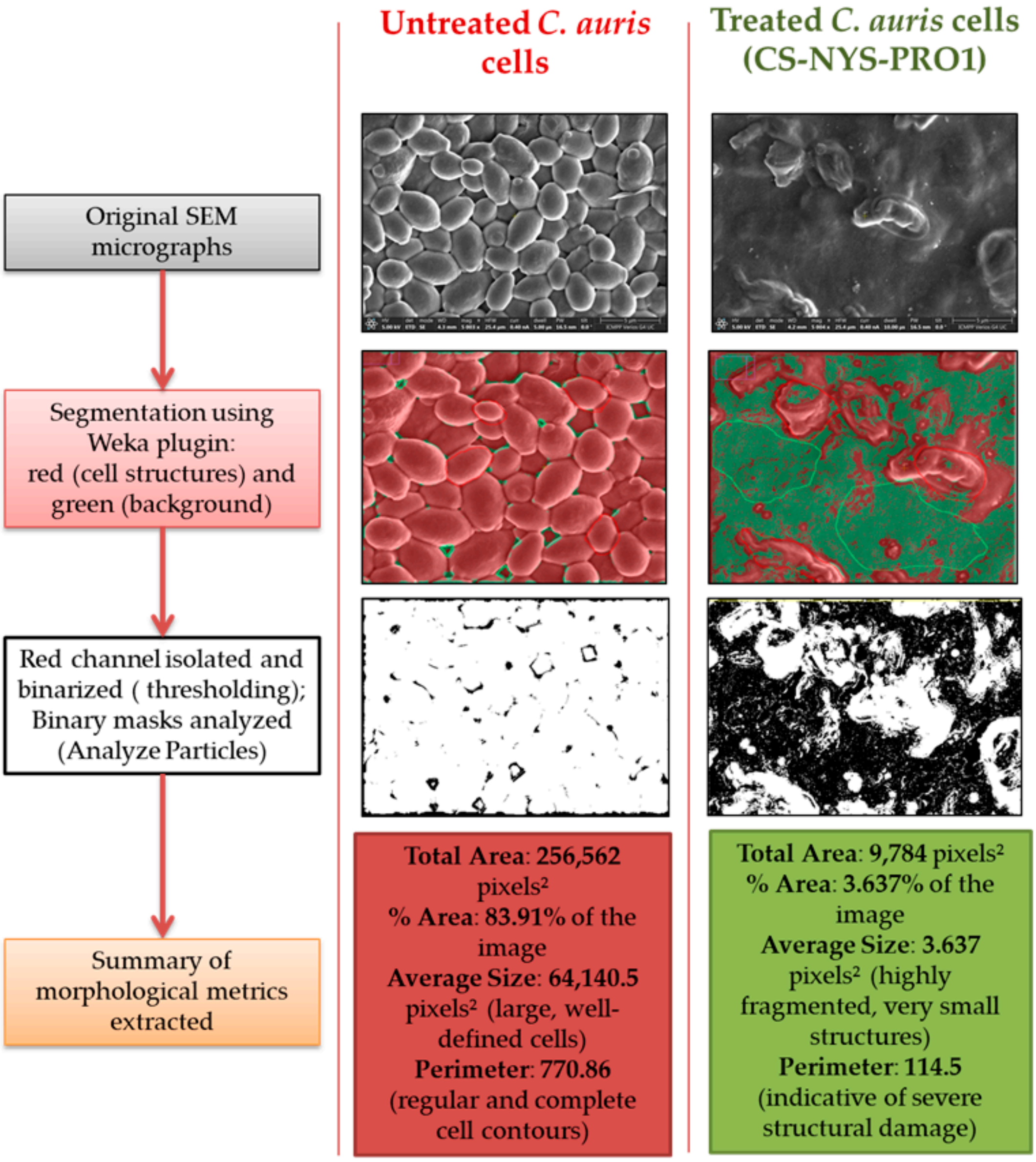

Figure S2. Workflow of SEM image processing and quantitative analysis using Trainable Weka Segmentation and ImageJ

Quantitative cell reduction calculation:

The percentage reduction in cell-covered area following treatment with CS-NYS-PRO1 hydrogel was calculated using the formula:

$$\text{Percentage Reduction} = 100 \times \text{Area}_{untreated} - \text{Area}_{treated} / \text{Area}_{untreated} \quad \text{(S1)}$$

$$\text{Percentage Reduction} = 100 \times (83.91\text{-}3.64) / 83.91 = 95.66\%$$

Table S2. Interaction descriptors from in silico molecular docking between the ligand (**nystatin molecule)** and the receptor (PDB ID: **5FSA)**.

Contacting residues in the Receptor	Specific interactions (number)	E_b (kcal/mol)	K_d (µM)
ASN^{187}, MET^{189}, LYS^{190}, GLU^{194}, PHE^{213}, ARG^{215}, SER^{216}, ALA^{218}, GLN^{219}, SER^{222}, ASP^{225}, LYS^{226}, GLY^{227}, PHE^{228}, THR^{229}, PRO^{230}, ILE^{231}, HIS^{310}, ASP^{504}, SER^{507}, MET^{508}, LEU^{511}, PRO^{512}, THR^{513}, GLU^{514}	hydrophobic contacts (**22**) hydrogen bonds (**3**)	– 9.44	0.121

Note about: Hydrogen Bond Analysis

Atom O **LYS 226** A accepts a bond from N NYS 1 N, O -H distance is 2.22 A, bond energy is 3.27 kcal/mol.

Atom OD2 **ASP 504** A accepts a bond from O NYS 1 N, O -H distance is 2.31 A, bond energy is 1.87 kcal/mol.

Atom OG **SER 507** A accepts a bond from O NYS 1 N, O -H distance is 2.41 A, bond energy is 1.94 kcal/mol.

3 hydrogen bonds: 3 accepted, 0 donated. Total hydrogen bond energy is 7.09 kcal/mol.

Table S3. Main characteristics of the receptor (crystal structure of dihydrofolate reductase (DHFR) from the emerging pathogenic fungus *Candida auris* (PDB ID: 7ZZX)), determined through structural analysis using YASARA Structure software.

**Characteristics**	**Value**
Number of amino acid residues	202
Molecular weight (kDa)	23.382
Radius of gyration R_g (Å)	16.84
Solvent accessible surface *SAS* (Å^2)	10,572
Primary structure content / Amino acid composition (codes):	*Count (relative frequency)*
Lysine (Lys, K)	18 (8.9%)
Arginine (Arg, R)	11 (5.4%)
Glutamic acid (Glu, E)	11 (5.4%)
Aspartic acid (Asp, D)	18 (8.9%)
Threonine (Thr, T)	6 (2.9%)
Histidine (His, H)	5 (2.5%)
Alanine (Ala, A)	8 (3.9%)
Serine (Ser, S)	14 (6.9%)
Methionine (Met, M)	7 (3.5%)
Proline (Pro, P)	8 (3.9%)
Glycine (Gly, G)	13 (6.4%)
Valine (Val, V)	13 (6.4%)
Cysteine (Cys, C)	0 (0%)
Tyrosine (Tyr, Y)	7 (3.5%)
Asparagine (Asn, N)	9 (4.4%)
Isoleucine (Ile, I)	12 (5.9%)
Leucine (Leu, L)	22 (10.9%)
Tryptophan (Trp, W)	5 (2.5%)
Glutamine (Gln, Q)	6 (2.9%)
Phenylalanine (Phe, F)	9 (4.4%)
Sum of positively charged residues, Σ (Lys^+ + Arg^+)	+29
Sum of negatively charged residues, Σ (Glu^- + Asp^-)	−29
Net charge of the Receptor	0.0
Secondary structure content:	*(relative frequency)*
α-helix	23.3%
β-sheet	36.6%
turn	13.9%
random coil	26.2%
3_{10}-helix	0.0%
π-helix	0.0%
